# Role of autophagy-related protein expression in patients with rectal cancer treated with neoadjuvant chemoradiotherapy

**DOI:** 10.1186/s12885-016-2250-0

**Published:** 2016-03-10

**Authors:** Byoung Yong Shim, Der Sheng Sun, Hye Sung Won, Myung Ah Lee, Soon Uk Hong, Ji-Han Jung, Hyeon-Min Cho, Yoon Ho Ko

**Affiliations:** Division of Oncology, Department of Internal Medicine, College of Medicine, The Catholic University of Korea, Seoul, Republic of Korea; Cancer Research Institute, College of Medicine, The Catholic University of Korea, Seoul, Republic of Korea; Department of Pathology, College of Medicine, Chung-Ang University, Seoul, Republic of Korea; Department of Hospital Pathology, College of Medicine, The Catholic University of Korea, Seoul, Republic of Korea; Department of General Surgery, College of Medicine, The Catholic University of Korea, Seoul, Republic of Korea

**Keywords:** Rectal cancer, Neoadjuvant chemoradiotherapy, Autophagy, LC3β, Beclin-1, Prognosis

## Abstract

**Background:**

Autophagy, a cellular degradation process, has complex roles in tumourigenesis and resistance to cancer treatment in humans. The aim of this study was to explore the expression levels of autophagy-related proteins in patients with rectal cancer and evaluate their clinical role in the neoadjuvant chemoradiotherapy setting.

**Methods:**

All specimens evaluated were obtained from 101 patients with colorectal cancer who had undergone neoadjuvant chemoradiotherapy and curative surgery. The primary outcomes measured were the expression levels of two autophagy-related proteins (microtubule-associated protein 1 light chain 3 beta (LC3β) and beclin-1) by immunohistochemistry and their association with clinicopathological parameters and patient survival.

**Results:**

Among the 101 patients, the frequency of high expression of beclin-1 was 31.7 % (32/101) and that of LC3β was 46.5 % (47/101). A pathologic complete response was inversely associated with LC3β expression (*P* = 0.003) and alterations in the expression of autophagy-related proteins (*P* = 0.046). In the multivariate analysis, however, autophagy-related protein expression did not show prognostic significance for relapse-free survival or overall survival.

**Conclusions:**

High expression of autophagy-related proteins shows a strong negative association with the efficacy of neoadjuvant chemoradiotherapy in patients with rectal cancer. Autophagy has clear implications as a therapeutic target with which to improve the efficacy of neoadjuvant chemoradiotherapy.

## Background

In an effort to improve local control and patient survival after surgical resection of rectal cancer, a multimodal treatment strategy, particularly involving neoadjuvant chemoradiotherapy, has been widely considered to be the standard treatment of localized rectal cancer [1]. After standard neoadjuvant chemoradiotherapy using 5-fluorouracil (5-FU)-based regimens, the pathologic complete response (ypCR) rate is approximately 8–15 %. In 20 % of patients, however, the response is poor or absent [2, 3]. Because multimodal treatment strategies are associated with substantial mortality, significant morbidity, and lifelong sequelae that may permanently impair quality of life, proper selection of patients for aggressive treatment is warranted. If the tumor response could be predicted before treatment, patients with a priori resistant tumors could be spared from radiation and undergo surgery without delay. Thus, identification of responders and nonresponders to chemoradiotherapy before surgery is surely of considerable clinical relevance. Several studies have evaluated the usefulness of clinical and pathological biomarkers in predicting the response to neoadjuvant chemoradiotherapy [4, 5], but their findings are unclear and controversial.

Autophagy, type II programmed cell death, is a highly regulated process that is usually activated in response to adverse environments. During autophagy, cytoplasmic materials are enclosed in double membrane-bound vesicles (autophagosomes) that are then targeted by lysosomes for degradation. Autophagy is a critical process that allows for cell conservation under stress conditions, including anticancer treatment. Several proteins are involved in the autophagy process; of these proteins, beclin-1 and light chain 3 (LC3) are key autophagy-related proteins. Beclin-1, the mammalian ortholog of yeast autophagy-related protein (Atg) 6, encoded by the *BECN1* gene, has a central role in several autophagy steps; its interaction with several cofactors induces initiation and nucleation of isolation during autophagy. During initiation of autophagy, substrates are trapped by autophagosomes that arise from the endoplasmic reticulum and trans-Golgi network. In addition, two ubiquitin-like conjugation reactions are essential for elongation of the phagophore membrane. These reactions involve the conjugation of several Atg proteins as well as the conjugation of microtubule-associated protein LC3 to phosphatidylethanolamine to form LC3β [6]. LC3, the mammalian homolog of yeast Atg8, is the most widely monitored autophagy-related protein [7].

The biological role of autophagy in cancer is controversial [6, 8]. Autophagy defects can accelerate tumorigenesis. The essential autophagy regulator *BECN1* is monoallelically deleted in many human ovarian, breast, and prostate cancers [9, 10]. However, other studies have suggested that autophagy promotes cell survival under stress conditions by degrading and recycling long-lived proteins and cellular components [11, 12]. A previous study demonstrated that autophagy is activated in colorectal cancer in vitro and in vivo and that autophagy may contribute to the survival of colorectal cancer cells that have acquired resistance to nutrient starvation [12]. The results of several studies of the prognostic roles of autophagy-related proteins are still conflicting [13–20]. These conflicting results could be due to the variable prognostic value of autophagy-related proteins, which depends on the intrinsic molecular heterogeneity of the tumor, the tumor stage, and the treatment regimen. Considering that chemotherapy and radiation disrupt the tumor architecture and vascularization, leaving any remaining tumor cells potentially vulnerable to adverse metabolic stress, autophagy may be crucial to tumor cell survival in patients undergoing anticancer treatment. Recent studies have suggested that tumor resistance to anticancer therapies, including radiation therapy, can be enhanced through upregulation of autophagy of colorectal cancer both in vitro [21] and in vivo [22, 23]. However, most preclinical experiments have utilized xenograft models, thereby eliminating the involvement of the innate immune system, which might play a critical role in determining the effectiveness of autophagy inhibition in chemosensitization or radiosensitization [24].

Thus, the aim of the present study was to clarify the clinical role of the expression of autophagy-related proteins (beclin-1 and LC3β) in the neoadjuvant setting for rectal cancer. We enrolled a homogenous cohort of patients who underwent neoadjuvant chemoradiotherapy and curative surgical resection, and we evaluated the expression of autophagy-related proteins in terms of their relationship with clinicopathological parameters and clinical outcomes.

## Methods

### Patients and specimens

We reviewed the clinical and pathological data of patients who were diagnosed with rectal cancer and underwent neoadjuvant chemoradiotherapy and laparoscopic surgery at St. Vincent’s Hospital of the Catholic University of Korea from 2005 to 2008. The inclusion criteria were: (i) a pathologically confirmed diagnosis of adenocarcinoma; (ii) neoadjuvant treatment with 50.4 Gy (1.8 Gy/day in 28 fractions) over 5.5 weeks, plus boluses of 5-FU (425 mg/m^2^/day) and leucovorin (20 mg/m^2^/day) on days 1–5 and 29–33, and surgery performed 7–10 weeks after completion of all therapies; (iii) follow-up for at least 2 years for patients with initial clinical stage II or III rectal cancer; (iv) more than near-complete total mesorectal excision (TME); and (v) available paraffin blocks of tumor specimens. The initial work-up before neoadjuvant chemoradiotherapy included a detailed clinical history and careful physical examination, determination of the Eastern Cooperative Oncology Group performance status, and assessment of hematological and biochemical profiles. Disease extension was assessed by computed tomography scans of the chest and abdomen, positron emission tomography–computed tomography, pelvic magnetic resonance imaging, and endorectal ultrasound. The images were independently reviewed by a radiologist blinded to the clinical information, and the pathologic findings were reviewed by two independent pathologists. Downstaging was defined as a staging reduction from the pretreatment clinical stage (cStage) to a pathologic stage (ypStage) (i.e., cIII to ypII, ypI, or yp0; cII to ypI or yp0). The pathologic response to chemoradiotherapy was reviewed and scored as follows: Grade 0, no response; Grade 1, necrosis or disappearance of tumor cells in less than 2/3 of the tumor; Grade 2, necrosis or disappearance of tumor cells in more than 2/3 of the tumor; and Grade 3, no viable cells (ypCR). The initial clinical and postoperative pathological staging was performed according to the staging criteria of the American Joint Committee on Cancer (AJCC) staging criteria, 7th edition. Informed consent for tissue samples was obtained at diagnosis and this study was approved by the Institutional Research Ethics Board of St. Vincent’s Hospital of the Catholic University of Korea.

### Immunohistochemical analysis

Immunohistochemical staining was performed using formalin-fixed, paraffin-embedded tissue samples from initial colonoscopic biopsies to examine the expression of LC3β and beclin-1 proteins. Immunohistochemistry was performed on 4-μm sections from the tissue microarray blocks using an autostainer (LabVision Autostainer LV-1; LabVision/Neomarkers, Fremont, CA) according to the manufacturer’s protocol. Tissue sections were mounted on superfrost glass slides, deparaffinized, and rehydrated through xylene and serial alcohol solutions. For antigen retrieval, the slides were immersed in 0.01 M citrate buffer (pH 6.0) by heating the sample in a pretreatment system for optimization of staining consistency (PT Link; Dako, Glostrup, Denmark) at a preheated temperature of 65 °C for holding and a targeted final temperature of 95 °C for 20 min. Tissue sections were treated with 0.3 % hydrogen peroxide in methanol for 30 min to block endogenous peroxidase activity. Rabbit polyclonal antibodies to LC3β and beclin-1 were purchased from Abcam (Cambridge, UK) and used at the following dilutions: beclin-1 (1:130) and LC3β (1:200). The tissue sections were then incubated with primary antibodies at room temperature for 24 h. Immunoreactions were detected by a conventional labeled streptavidin-biotin method (LSAB2 System-HRP; Dako). The color reaction was completed by a 5-min incubation with 3,3’-diaminobenzidine, and hematoxylin counterstaining was used. The results were analyzed by one pathologist (S.U.H.) who was blinded to all patients’ clinical data. Immunostaining was interpreted using a semiquantitative histologic score. The staining intensity was scored as no staining (0), weak staining (1+), moderate staining (2+), or strong staining (3+). The percentage of stained area was classified as follows: 0, 0–10 %; 1, 11–25 %; 2, 26–50 % and 3, 51–100 %. The intensity and percentage scores were multiplied to yield a composite score of 0 to 9 for each specimen. High or low protein expression was defined based on the median composite score of each protein (LC3β, 0–2 vs. 3–9; beclin-1, 0–6 vs 7–9).

### Statistical analysis

The overall survival (OS) duration was calculated from the date of diagnosis to the date of death or last follow-up visit. The relapse-free survival (RFS) duration was calculated from the date of diagnosis to the date of first distant or local disease recurrence or last follow-up. The Kaplan–Meier method was used to analyze “time-to-event” data, and the significance of differences in the cumulative survival curves were evaluated using the log-rank test. Cox proportional hazards regression models were used to investigate the significance of prognostic factors. Autophagy-related proteins and all variables with a *P* value of <0.2 in the univariate analysis were included in the multivariate analysis. Correlations between immunohistochemical profiles and clinicopathological variables were analyzed by the chi-squared or Fisher’s exact test. Comparisons of immunohistochemical expression were performed with an independent-samples *t*-test for continuous variables. Survival rates and hazard ratios (HRs) are presented with their respective 95 % confidence intervals (CIs). A P value of <0.05 was considered to indicate statistical significance. All statistical analyses were performed using the R statistical software package (R Foundation for Statistical Computing, Vienna, Austria).

## Results

### Patients’ clinical characteristics

In total, 101 paraffin blocks of tumor samples were available from patients who had undergone neoadjuvant chemoradiotherapy and laparoscopic TME. The clinical and pathological characteristics of the cohort are shown in Table 1. The patient cohort comprised 69 men and 32 women with a median age of 62 years (range, 36–83 years). According to the AJCC staging criteria, 52 (51.5 %) patients had stage II disease and 49 (48.5 %) had stage III. Downstaging after neoadjuvant chemoradiotherapy resulted in a change of 26 (50.0 %) of 52 patients with stage cII disease to ypI (*n* = 16) and yp0 (*n* = 10), and 31 (63.3 %) of 49 patients with stage cIII disease to ypII (*n* = 13), ypI (*n* = 5), and yp0 (*n* = 13). Overall, the rate of downstaging of this preoperative therapy was 56.4 % (*n* = 57), and the rate of ypCR was 22.8 % (*n* = 23) (Table 2). The median follow-up duration was 51.0 months (range, 13–84 months) after the initial pathological diagnosis. Among the 101 patients, 20 (19.8 %) died of their tumors and 81 (80.2 %) were still alive at the last follow-up. The overall recurrence rate was 31.7 % (*n* = 32).

**Table 1:** Baseline clinicopathological characteristics of 101 patients with rectal cancer

	Total	
	Patients (n)	%
Age in years, median (range)	62 (36-83)	
Sex		
Male	69	68.3
Female	32	31.7
ECOG performance status		
0	45	44.6
1	56	55.4
Clinical T stage		
cT2	2	2.0
cT3	96	95.0
cT4	3	3.0
Clinical N stage		
cN0	52	51.5
cN1	32	31.7
cN2	17	16.8
Clinical TNM stage		
cII	52	51.5
cIII	49	48.5
Histological differentiation		
Well	18	17.8
Moderately	76	75.2
Poorly	7	6.9
CEA in ng/ml, median (range)	3.28 (0.67-205.64)	
LDH in IU/L, median (range)	288.5 (114.0-582.0)	

*ECOG* Eastern Cooperative Oncology Group, *CEA* carcinoembryonic antigen, *LDH* lactate dehydrogenase

**Table 2:** Treatment response to neoadjuvant chemoradiotherapy

Characteristic	Patients, n (%)
Pathologic T stage	
ypT0	23 (22.8)
ypT1	5 (5.0)
ypT2	22 (21.8)
ypT3	49 (48.5)
ypT4	2 (2.0)
Pathologic N stage	
ypN0	71 (70.3)
ypN1	24 (23.8)
ypN2	6 (5.9)
Pathologic TNM Stage	
No residual tumor	23 (22.8)
I	21 (20.8)
II	27 (26.7)
III	30 (29.7)
Pathologic response evaluation	
Grade 0: No change	19 (18.8)
Grade 1: Necrosis in less than 2/3 of the tumor	33 (32.7)
Grade 2: Necrosis in more than 2/3 of the tumor	26 (25.7)
Grade 3: No viable cells	23 (22.8)
Downstaging	57 (56.4)
ypCR	23 (22.8)

*ypCR* pathologic complete response

### Immunohistochemical staining patterns and relationship with clinicopathological findings

**Fig. 1:**
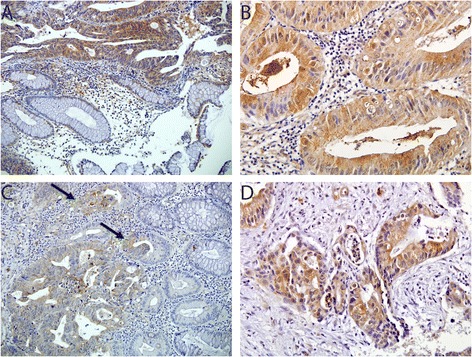
Immunohistochemical staining for beclin-1 and LC3β. **a** LC3β expression is also noted in adenocarcinoma, in contrast to its absence in normal crypts (×200). **b** LC3β is strongly positive for invasive adenocarcinoma (×400). **c** Beclin-1 expression is observed in invasive adenocarcinoma and crypts with partial cancerization (*arrow*), whereas normal crypts are negative for beclin-1 (×200). **d** Strong cytoplasmic expression for belcin-1 in invasive adenocarcinoma (×400)

**Fig. 2:**
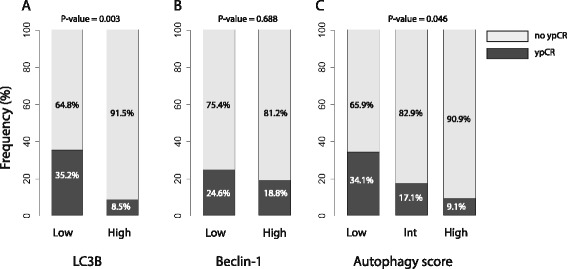
Relationship between autophagy-related protein expression and pathologic complete response (ypCR). ypCR is found more frequently in **a** rectal cancer with low LC3β expression (*P* = 0.003) and **c** low autophagy-related protein expression (*P* = 0.046), whereas **b** the expression of beclin-1 is not associated with the achievement of ypCR (*P* = 0.688)

Figure 1 shows a representative immunohistochemistry result. Of the 101 patients, LC3β and beclin-1 protein expression were detected in 79.2 % (80/101) and 87.1 % (88/101) patients, respectively. The frequency of high expression of beclin-1 was 31.7 % (32/101), and that of LC3β was 46.5 % (47/101). The expression of LC3β and beclin-1 in tumor cells was predominantly localized to the cytoplasm, in contrast to their absence in normal crypts. Associations between autophagy-related protein expression and clinicopathological features, including well-known prognostic factors such as pathologic stage, lymph node metastasis, histologic grade, serologic tumor marker levels, and post-chemoradiotherapy pathologic features, were also explored (Table 3). Low LC3β expression was significantly correlated with ypCR (*P* = 0.003, Fisher’s exact test) (Fig. 2a). Although the number of patients with stage cT4 tumors (*n* = 3) was small, high beclin-1 expression was significantly correlated with a more advanced clinical T stage (*P* = 0.030, Fisher’s exact test). However, no significant correlation was observed between high expression of autophagy-related proteins and the other clinicopathological parameters, including ypCR (Fig. 2b). Because the associations between the expression levels of the autophagy-related proteins were strong and significant (*P* = 0.005, chi-squared test), consistent with their known roles in regulating autophagy activation, we divided the patients into three groups according to the number of changes in their expression: low (*n* = 44, 43.6 %), intermediate (*n* = 35, 34.7 %), and high (*n* = 22, 21.8 %) groups. Considering the clinicopathological differences according to these autophagy scores, a lower number of changes was found to be significantly associated with a higher achievement of ypCR: low (15/44, 34.1 %), intermediate (6/35, 17.1 %), and high autophagy scores (2/22, 9.1 %; *P* = 0.046, Fisher’s exact test) (Fig. 2c).

**Table 3:** Clinicopathological factors and their relationship to the expression of autophagy proteins

	LC3β		Beclin-1	
	Low (%)	High (%)	Low (%)	High (%)
No. patients (%)	54 (53.5)	47 (46.5)	69 (68.3)	32 (31.7)
Clinical T stage				
cT2-3	53 (98.1)	45 (95.7)	69 (100)	29 (90.6)
cT4	1 (1.9)	2 (4.3)	0 (0)	3 (9.4)
P value		0.596		0.030
Clinical N stage				
cN0	26 (48.1)	26 (55.3)	34 (49.3)	18 (56.3)
cN1-2	28 (51.9)	21 (44.7)	35 (50.7)	14 (43.7)
P value		0.603		0.661
Grade				
Well/moderately	49 (90.7)	42 (95.5)	62 (89.9)	32 (100)
Poorly	5 (9.3)	2 (4.5)	7 (10.1)	0 (0)
P value		0.444		0.094
ypCR				
Yes	19 (35.2)	4 (8.5)	17 (24.6)	6 (18.8)
No	35 (64.8)	43 (91.5)	52 (75.4)	26 (81.2)
P value		0.003		0.688

*ypCR* pathologic complete response

### Survival analysis with respect to autophagy-related proteins

**Fig. 3:**
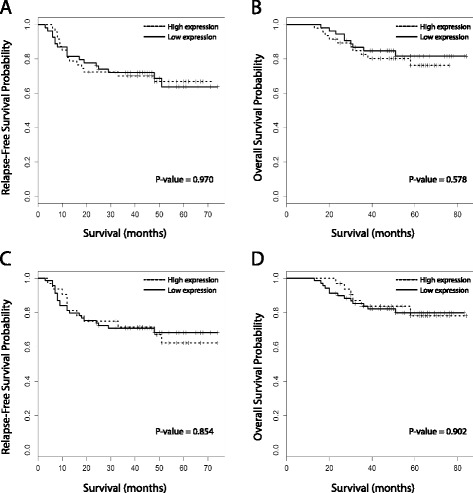
Survival curves of patients with rectal cancer as stratified by the expression of the autophagy-related proteins LC3β (**a**, relapse-free survival; **b**, overall survival) and beclin-1 (**c**, relapse-free survival; **d**, overall survival)

The 5-year OS rate and RFS rate for patients who had undergone resection of rectal cancer was 78.9 and 65.7 %, respectively. Based on the univariate analysis, predictors of RFS included cT stage (*P* < 0.001), cN stage (*P* = 0.050), histological differentiation (*P* = 0.0104), and the achievement of ypCR (*P* = 0.050) (Table 4). Kaplan–Meier survival curves did not reveal a significant association between high levels of LC3β or beclin-1 and disease recurrence (*P* = 0.970 and *P* = 0.854, respectively) (Fig. 3a). In the multivariate analysis for RFS, significant predictors were advanced cT stage (HR, 7.236; 95 % CI, 2.20–15.24; P < 0.001), the clinical lymph node metastasis status (HR, 2.257; 95 % CI, 2.81–18.62; *P* = 0.022), high-grade tumor differentiation (HR, 5.783; 95 % CI, 1.13 – 4.52; *P* < 0.001), and ypCR (HR, 0.216; 95 % CI, 0.07 – 0.64; *P* = 0.006). However, no significant association was observed between LC3β or beclin-1 expression and disease recurrence (Table 4; Fig. 3a and c). Univariate analysis for OS revealed that the following factors were significantly correlated with OS: advanced cT stage (*P* = 0.036), clinical regional lymph node metastasis (*P* = 0.031), and pathologic response (*P* = 0.038). Neither LC3β nor beclin-1 expression was correlated with OS (Table 5). In the multivariate analysis, clinical lymph node metastasis (HR, 4.572; 95 % CI, 1.33 – 15.71; *P* = 0.016), high-grade tumor differentiation (HR, 12.264; 95 % CI, 2.06 –73.14; *P* = 0.006), and pathologic response (HR, 0.176; 95 % CI, 0.04 – 0.80; *P* = 0.025) were found to be significant prognostic factors. The expression of any markers, however, was not significantly correlated with OS (Table 5; Fig. 3b and d).

**Table 4:** Univariate and multivariate relapse-free survival analysis of patients who underwent rectal cancer resection (Cox proportional hazard model)

	Variable	Univariate analysis			Multivariate analysis		
		HR	95 % CI	P value	HR	95 % CI	P value
Age		0.995	0.97 – 1.03	0.777			
Sex	Female vs. Male	0.931	0.44 – 1.97	0.852			
cT stage	cT4 vs. cT2-3	7.920	2.33 – 27.00	<0.001	7.236	2.20 – 15.24	< 0.001
cN stage	cN1-2 vs. cN0	2.050	1.00 – 4.19	0.050	2.257	2.81 – 18.62	0.022
Histological grade	Poorly vs. Well/Mod	3.520	1.34 - 9.20	0.010	5.783	1.13 – 4.52	< 0.001
CEA		1	0.99 – 1.01	0.482			
LDH		1	0.99 – 1.00	0.939			
ypCR	Yes vs. Nos	0.308	0.09 –1.01	0.050	0.216	0.07 – 0.64	0.006
LC3β expression	High vs. Low	0.986	0.49 – 1.98	0.970	0.666	0.27 – 1.67	0.386
Beclin-1 expression	High vs. Low	1.070	0.52 – 2.23	0.854	1.147	0.43 – 3.09	0.786

*HR* hazard ratio, *CI* confidence interval, *CEA* carcinoembryonic antigen, *LDH* lactate dehydrogenase, *cT* clinical T stage, *cN* clinical N stage, *ypCR* pathologic complete response

**Table 5:** Univariate and multivariate overall survival analysis of patients who underwent rectal cancer resection (Cox proportional hazard model)

	Variable	Univariate analysis			Multivariate analysis		
		HR	95 % CI	P value	HR	95 % CI	P value
Age		1.010	0.97 – 1.06	0.521			
Sex	Female vs. Male	0.489	0.16 – 1.48	0.206	0.379	0.12 – 1.16	0.088
cT stage	cT4 vs. cT2-3	4.840	1.11 – 21.1	0.036	3.305	0.72 – 15.15	0.124
cN stage	cN1-2 vs. cN0	3.070	1.11 – 8.54	0.031	4.572	1.33 – 15.71	0.016
Histological grade	Poorly vs. Well/Mod	2.870	0.84 – 9.86	0.094	12.264	2.06 – 73.14	0.006
CEA		1.010	1.00 – 1.02	0.135	1.007	1.00 – 1.02	0.203
LDH		0.999	0.99 – 1.01	0.719			
ypCR	Yes vs. Nos	0.338	0.12 – 0.94	0.038	0.176	0.04 – 0.80	0.025
LC3β expression	High vs. Low	1.290	0.52 – 3.17	0.581	1.460	0.60 – 3.58	0.409
Beclin-1 expression	High vs. Low	0.942	0.36 – 2.48	0.904	0.773	0.23 – 2.58	0.675

*HR* hazard ratio, *CI* confidence interval, *CEA* carcinoembryonic antigen, *LDH* lactate dehydrogenase, *cT* clinical T stage, *cN* clinical N stage

## Discussion

Neoadjuvant chemoradiotherapy and TME are the standard treatment for patients with locally advanced rectal cancer [1]. The response to neoadjuvant chemoradiotherapy is known to be an indicator of the clinical outcomes in patients with rectal cancer, and several biomarkers that can predict the response to neoadjuvant therapy in patients with rectal cancer have been investigated using immunohistochemical approaches. However, the clinical role of autophagy in the neoadjuvant setting for rectal cancer has not yet been discussed. To the best of our knowledge, the current report is the first study to demonstrate the clinical role of autophagy-related protein expression in a homogenous cohort of patients with rectal cancer who underwent neoadjuvant chemoradiotherapy and laparoscopic TME. In the present study, we showed that high expression of autophagy-related proteins was associated with decreased ypCR, but the expression of these proteins was not associated with survival outcomes in patients who underwent neoadjuvant chemoradiotherapy. These results support those of previous studies of the contribution of autophagy to radioresistance in cancer cell [21, 25], suggesting that autophagy expression may be strongly predictive of a poor response to neoadjuvant chemoradiotherapy.

Previous studies have shown that autophagy-related proteins are present in colorectal cancer as assessed by an LC3β protein expression level of 74 % to almost 100 % and by a beclin-1 protein expression level of 71–83 % [13, 17, 18], which is consistent with the findings of the present study. The role of autophagy in tumorigenesis and tumor progression remains debatable; there is evidence that autophagy may contribute to tumor suppression, whereas other evidence shows that it is clearly oncogenic in some contexts. Hemizygosity (*BECN 1*^+/-^) of *BECN1* or knockout of the gene encoding the UVRAG-binding protein BIF-1 (Bax-interacting protein-1) leads to a high incidence of different spectrums of tumor types in mice [26, 27]. In addition, ectopic expression of the *BECN1* gene or UVRAG have both been shown to suppress the growth of xenografts of human colon cancer cell lines [28, 29], suggesting that autophagy plays a tumor-suppressive role in tumorigenesis. In line with the results of these studies, lower LC3 or beclin-1 protein expression has also been found in cancer cells than in adjacent normal tissues in various human malignancies, including ovarian cancers [30], breast cancers [31], and esophageal cancers [32]. In contrast, an increasing body of evidence supports the notion that tumorigenesis can be promoted by the prosurvival function of autophagy under stressful conditions. Consistent with this concept, autophagy inhibition either by genetic silencing of autophagy-associated genes (such as *BECN1*, *ATG*5, *ATG7*, or *ATG12*) or by the use of pharmacological inhibitors (such as chloroquine, an inhibitor of autophagosome–lysosome fusion and lysosomal acidification) has been shown to enhance cell death of growth factor–starved cells in which apoptosis has been genetically ablated [11, 33]. In the major gastrointestinal cancers (such as esophageal, stomach, and colorectal cancers), autophagy activity is reportedly activated in contrast to their normal counterparts [7, 12, 13]. Sato et al. indicated that autophagy might provide an alternative source of energy through degradation of its organelles in colorectal cancer cell lines resistant to nutrient-deprivation culture conditions; they also showed strong LC3β expression in 59 of 80 colorectal cancer specimens at different stages (73.8 %) and in 0 of 65 samples of normal colorectal mucosa by immunohistochemistry and western blotting [12], similar to our data. These results suggest that autophagy may contribute to tumor progression in colorectal cancer. Colorectal cancer is, however, a heterogeneous disease in terms of its clinical behavior and molecular profile [34], and previous studies have found considerable variation in the stage and treatments [13–15, 17–20, 35]. Thus, our results cannot be considered to be representative of all patients with rectal cancer.

In the present study, the LC3β expression level was not associated with age, sex, primary disease stage (tumor size and node status), histologic grade, or serum tumor markers. However, low LC3β protein expression was remarkably associated with the achievement of ypCR and pathologic T0 stage after neoadjuvant chemoradiotherapy. We also found a highly consistent correlation between the autophagy score and the achievement of ypCR. These findings are in accordance with a recent study by Tougeron et al. [36]. In 96 cases of rectal carcinoma, increased beclin-1 expression predicted a significantly reduced pathological response (macroscopic versus microscopic or no residual tumor) to chemoradiation (high: 14.2 % vs. low: 40 %; *P* = 0.017). In addition, Guo et al. demonstrated that patients with low LC3 expression had a higher objective response rate among patients with advanced colorectal cancer treated with cetuximab-containing chemotherapy [35]. These findings of the impact of autophagy activity on the response to cancer treatment are well supported by in vitro and in vivo studies. A previous study demonstrated that tumor resistance to anticancer therapies, including chemotherapy and radiation therapy, could be enhanced through upregulation of autophagy using various types of tumor cell lines [23]. The use of the autophagy inhibitors chloroquine and 3-methyladenine inhibitor leads to significant 5-FU-induced inhibition of colorectal cancer growth [22, 37, 38]. Apel et al. demonstrated that antagonization of *BECN1* using specific antisense oligonucleotides in vitro reduced irradiation-induced accumulation of autophagosomes, and short-time inhibition of autophagy along with radiotherapy led to enhanced cytotoxicity of radiotherapy in various types of resistant cancer cell [21]. Additionally, in a recent experimental study of colorectal cancer cell lines, autophagy inhibition by chloroquine also increased sensitivity to concurrent treatment with 5-FU and radiation [21]. Thus, we could speculate that the pretreatment autophagy status of tumor cells might predict the efficacy of chemoradiotherapy in the neoadjuvant setting for patients with rectal cancer.

The expression of autophagy-related proteins (particularly LC3 and beclin-1) is reportedly a prognostic factor in several types of solid tumors, but the results are conflicting [39–41]. The prognostic significance of LC3β or beclin-1 for colorectal cancer is also contradictory [14, 18, 20]. High “stone-like” intracellular structure counts of LC3α expression, presumably reflecting an excessive autophagic response, were associated with tumor hypoxia and poor clinical outcomes [15]. Koukourakis et al. documented that loss of beclin-1 expression defines a poor prognosis presumably by promoting antiapoptotic pathways, while overexpression of the protein, being linked with tumor hypoxia and acidity, also defines subgroups of tumors with aggressive clinical behavior [14]. A recent meta-analysis showed that elevated beclin-1 expression is associated with tumor metastasis and a poor prognosis in patients with colorectal cancer [19]. The prognostic role of the expression of autophagy-related proteins in rectal cancer, however, is unclear because of heterogeneity due to the mixture of colon and rectal cancers and differences in the stages and treatment modalities among the studies.

Autophagy is regulated by several signal transduction pathways, including the PI3K/AKT/mTOR pathway, Bcl-2 family, RAS pathway, and p53 [8, 42]. Mutations in *TP53* and *KRAS* are common genetic alterations in patients with colorectal cancer. The tumor suppressor p53 protein can modulate autophagy depending on its cellular localization; when nuclear, it enhances transcription of pro-autophagic genes, whereas when cytoplasmic, it inhibits transcription through various mechanisms of the autophagic process [43, 44]. Defective p53 results in autophagy induction and chemoresistance to chemotherapeutic agents in colon cancer cells [45], and inhibition of autophagy enhances the sensitivity of colon cancer cells with wild-type p53 to chemotherapeutic agents [46]. The K-RAS oncogene is known to induce autophagy, and autophagy is activated constitutively and essential in oncogenic Ras-driven tumourigenesis [16, 47]. Thus, when investigating autophagy in patients with colorectal cancer, genetic alterations should be considered. Further studies of the relationship between p53 or K-RAS mutation and the autophagic pathway in patients with colorectal cancer are needed.

## Conclusion

The small sample size, small diagnostic tissue samples, and retrospective nature of the present study might not reflect the biological properties of the entire population with rectal cancer and does not allow us to draw conclusions regarding the prognostic value of LC3β or beclin-1 in rectal cancer. However, the present study has suggested that high expression of autophagy-related proteins has a negative association with the efficacy of neoadjuvant chemoradiotherapy in patients with rectal cancer, and autophagy may have clear implications as a therapeutic target with which to improve the efficacy of neoadjuvant chemoradiotherapy. A phase II trial is ongoing to determine whether combining hydroxychloroquine together with capecitabine, oxaliplatin, and bevacizumab is effective in treating patients with metastatic colorectal cancer [48]. Autophagy inhibition may be a promising therapeutic strategy with which to overcome the resistance of chemotherapy and radiotherapy. In addition, well-designed prospective randomized studies are needed to clarify the role of autophagy in rectal cancer patients with preoperative chemoradiotherapy.

## Abbreviations

5-FU: 5-fluorouracil
AJCC: the American Joint Committee on Cancer
Atg: autophagy-related protein
BIF-1: Bax-interacting protein-1
Cis: confidence intervals
cStage: pretreatment clinical stage
HRs: hazard ratios
LC3β: microtubule-associated protein 1 light chain 3 beta
OS: overall survival
RFS: relapse-free survival
TME: total mesorectal excision
ypCR: the pathologic complete
ypStage: pathologic stage
